# Significance of Preoperative Total Lymphocyte Count as a Prognostic Criterion in Adult Cardiac Surgery

**DOI:** 10.5812/aapm.20331

**Published:** 2014-06-23

**Authors:** Nahid Aghdaii, Rasoul Ferasatkish, Ali Mohammadzadeh Jouryabi, Seyed Hosein Hamidi

**Affiliations:** 1 Rajaei Cardiovascular Medical and Research Center, Iran University of Medical Sciences, Tehran, Iran

**Keywords:** Total Lymphocyte Count, Cardiopulmonary Bypass, Mortality, Morbidity, Postoperative Complications

## Abstract

**Background:**

Evaluation of operational risk is a consequential goal in perioperative management of patients in cardiac surgery. Preoperative total lymphocyte count (PTLC) is a prognostic criterion of adverse major cardiovascular outcomes.

**Objectives:**

The purpose of this study was to investigate the prognostic value of PTLC as an independent predictor of postoperative morbidity and mortality in cardiac surgery.

**Patients and Methods:**

Of 1604 patients scheduled for cardiac surgery between September, 2012 and March, 2013, a total of 1171 consecutive patients underwent elective primary valvular heart surgery and coronary artery bypass grafting. The patients were divided to three groups according to their PTLCs. The baseline characteristics and postoperative mortality and morbidity of the patients as well as the intensive care unit (ICU) stay according to the PTLCs were recorded and analyzed. The only inclusion criterion was a preoperative complete blood count. Exclusion criteria included: ages under 18 or over 80 years old, emergency surgery, adult patients with congenital heart disease and previous open heart surgery, and patients with any bacterial or viral infection during two weeks before the surgery. Protocol of anesthetic medications was used in all the patients similarly and according to standard. All the patients were admitted to the ICU after the surgery.

**Results:**

A PTLC < 1500 cells/µL was associated with significantly high mortality and morbidity (P = 0.0001). In-hospital mortality and major composite morbidity were 9.65% and 28.4%, respectively. Low PTLC was associated with more frequent need for inotropic and intra-aortic balloon pump (IABP) support (P < 0.001), dialysis-dependent acute renal failure (P = 0.0001), postoperative superficial wound infections (P = 0.0001) and prolong ICU stay (P = 0.0001).

**Conclusions:**

Our study results showed that low PTLC was an independent, valuable prognostic criterion, with high sensitivity and specificity for evaluation of postoperative morbidity and mortality in cardiac surgery.

## 1. Background

For many years, operative mortality was the sole criterion used for evaluation of patient outcomes and many studies analyzed the mortality of cardiac operations; but, the studies that concentrated on analysis of perioperative morbidity and its influence on global early and late outcomes are much fewer. It is clearly known that other nonfatal postoperative complications can significantly impact not only the perioperative period but also the patient’s quality of life (1, 2). Therefore, identification of risk factors for increased perioperative morbidity in cardiac surgery may provide valuable information, which may subsequently be used to improve the quality of care. The role of low-grade inflammation in pathogenesis of atherosclerosis and its acute complications has been well-recognized (3) and several biological markers of inflammation like albumin concentration (4), body mass index (BMI) (5) and C-reactive protein (6) predict cardiovascular risks (7). One of the most readily obtainable indices is the preoperative total lymphocyte count (PTLC), a simple inflammatory marker, which is found to be a significant independent predictor of adverse outcomes in patients with coronary artery disease (8-12). The prognostic role of PTLC has not been highly investigated in cardiac surgery, but it is known that PTLC is a significant predictor of mortality among patients who have underwent coronary artery bypass graft (CABG) (13-16) or other cardiac surgeries. It is known that lymphopenia is an unfavorable prognostic factor in oncology (17), patients with dialysis-dependent chronic renal disease (18), elderly patients with cervical hip fracture (19), patients with coronary atherosclerosis (CA) (10, 12), and ones with chronic or acute heart failure (20-23). Malnutrition (24), increased corticosteroid hormones, cortisol, and catecholamine in blood in response to a pathologic process (25), impairment of the microcirculation, and hypoxia, are responsible for reduction in amount and functional capacity of lymphocytes (26, 27). The prognostic role of PTLC has been investigated in noncardiac surgeries (12, 28, 29). However, its prognostic value remains undetermined. 

## 2. Objectives

The objective of this study was to investigate the prognostic value of TLC in survival of patients under cardiac surgery with cardiopulmonary bypass (CPB).

## 3. Patients and Methods

Of 1604 patients scheduled for cardiac surgery between September 23, 2012 and March 20, 2013, a total of 1171 consecutive patients (753 males and 418 females; age range: 18-80 years old) underwent elective primary cardiac surgery at Rajaei Cardiovascular Medical and Research Center. The study protocol was approved by the Division Ethics Committee of our institute as a retrospective observational study. The patients’ records regarding preoperative age, gender, BMI, ejection fraction, type of surgery, CPB time, white blood cell (WBC) lymphocyte, morbidity and mortality in hospital, and duration of ICU stay were stored in our database. The only inclusion criteria were a preoperative complete blood count and using standard and similar anesthetic medications in all of patients. Exclusion criteria included: ages below 18 or over 80 years old (n = 133), emergency surgery (n = 201), congenital heart disease (n = 56) or previous open heart surgery (n = 33), chronic inflammatory or autoimmune diseases, and bacterial or viral infection during two weeks before the surgery (n = 10). Demographic variables, some of coronary artery disease risk factors (diabetes), operative variables (CPB time), left ventricular ejection fraction, and postoperative mortality and morbidity rates were analyzed retrospectively (Tables 1 and 2). We obtained the left ventricular ejection fraction from echocardiography reports. To study the baseline characteristics according to PTLC, all analyses were conducted using blood samples obtained during 48 hours before the surgery. The patients were divided to three groups depending on their preoperative PTLCs; group one: ≤ 1000 cells/µL, group two: 1001-1500 cells/µL, group three: > 1500 cells/µL. In our study, the primary outcome measures were mortality and morbidity.

### 3.1. Type of Outcome Measures

Mortality was defined as in-hospital mortality and morbidity as organ failure immediately after surgery, which was likely secondary to the effects of surgery and cardiopulmonary bypass and resolved without affecting serious outcome. Conversely, organ failure after prolonged ICU stay was likely secondary to nosocomial infection and profoundly influenced the outcome. Three morbid postoperative events, either life threatening or potentially resulting in permanent functional disability, were analyzed, which were mediastinitis, renal failure, and cardiovascular failure. A model for composite morbidity (association of two or more major morbid events) was also developed. Except for mediastinitis, which was evaluated as an in-hospital event, morbidity complications were analyzed as events occurring during the ICU stay, unlimited in time. Postoperative complications were classified as cardiac (e.g. acute myocardial infarction based on electrocardiograms (ECG) and/or enzyme alterations, arrhythmias requiring treatment, pulmonary edema, congestive heart failure, low cardiac output syndromes, patients requiring post-bypass intra-aortic balloon pump or inotropic drugs, etc.) and noncardiac (eg, dialysis-dependent acute renal failure (ARF), liver insufficiency, gastrointestinal complications, cerebrovascular accidents, infectious complications, ICU stay more than three days defined as a prolong stay, etc.). Of 1171 patients operated on CPB under mild hypothermia, 674 underwent CABG surgery; 363 underwent valvular surgery (valvular replacement or valvular repair) and 134 underwent CABG in association with valvular surgery. Protocol of anesthetic medications was used in all of patients similarly and according to standard. Before the induction of anesthesia, all patients were premedicated with intramuscular lorazepam 1 mg and morphine sulfate 0.1 mg/kg, one hour before entering the operating room. Induction of anesthesia under ECG monitoring, pulse oximetry, invasive arterial blood pressure with etomidate 0.2 mg/kg, sufentanil 2.5 μg/kg and cisatracurium 0.2 mg/kg and maintenance of anesthesia after insertion of central venous line with continuous infusion of midazolam, sufentanil and atracurium, was performed in both groups. All patients underwent a full median sternotomy. Antegrade crystalloid or blood cardioplegia was used depending on the surgeon’s preferences. All the patients were admitted to the ICU after the surgery. When there was no evidence of bleeding and the patient was awake, cooperative and comfortable, cardiovascularly stable, normothermic, and with an acceptable blood gas on FIO_2_ ≤ 0.4, positive end-expiratory pressure ≤ 5 cm H_2_O, pressure support ≤ 10 cm H_2_O, tidal volume ≥ 5 mL/kg, and spontaneous respiratory rate < 20/minute, extubation was undertaken.

**Table 1.** Baseline Characteristics According to the Preoperative Total Lymphocyte Count ^a,b^

	PTLC ≤ 1000	1000 > PTLC < 1500	PTLC > 1500	P Value
**Number of patients**	26	380	765	
**Age, y**	52.62 ± 17.36	56.07 ± 15.30	56.71 ± 14.31	0.325
**Sex, male/female**	19/7	245/135	489/276	0.630
**BMI, kg/m** ^**2**^	22.54 ± 2.25	24.02 ± 2.50	24.89 ± 2.37	0.0001
**Surgery type**				
CABG	5 (0.7)	147 (21.8)	522 (77.4)	0.0001
Valvular	12 (3.3)	150 (41.3)	201 (55.4)	0.0001
Valvular + CABG	9 (6.9)	82 (62.6)	40 (30.5)	0.0001
**Mean LVEF, %**	32.5 ± 7.80	38.56 ± 7.41	46.58 ± 5.65	0.0001
**CPB, min**	230.04 ± 71.16	138.54 ± 30.9	78.82 ± 20.63	0.0001
**Diabetes**	14 (5.2)	203 (75.2)	53 (19.6)	0.0001
**Lymph, cells/µL**	922 ± 36.33	1312 ± 129.97	1846 ± 175.43	0.0001

^a^ Abbreviations: BMI, body mass index; LVEF, left ventricular ejection fraction; CABG, coronary artery bypass graft; CPB, cardiopulmonary bypass; PTLC: preoperative total lymphocyte count.

^b^ Data are presented as No. (%) or Mean ± SD.

**Table 2.** Postoperative Complications and Mortality According to the Preoperative Total Lymphocyte Count ^a,b^

	PTLC ≤ 1000	1000 > PTLC < 1500	PTLC > 1500	P Value
**Number of patients**	26	380	765	
**Inotropic support**	25 (8.2)	223 (73.1)	57 (18.7)	0.0001
**IABP support**	9 (18.4)	32 (65.3)	8 (16.3)	0.0001
**Infectious complications**	22 (14.6)	117 (77.5)	12 (7.9)	0.0001
**Dialysis-dependent ARF**	21 (38.2)	33 (60)	1 (1.8)	0.0001
**Morbidity**	25 (7.5)	247 (74.2)	61 (18.3)	0.0001
**Prolong ICU stay**	26 ± 0.27	378 ± 0.50	765 ± 0.25	0.0001
**Mortality**	18 (15.9)	78 (69)	17 (15)	0.0001

^a^ Abbreviations: ARF, acute renal failure; IABP, intra-aortic balloon pump; ICU, intensive care unit; PTLC, preoperative total lymphocyte count.

^b^ Data are presented as No. (%) or Mean ± SD.

### 3.2. Intervention

Data including preoperative demographics, comorbidity, routine laboratory testing, surgical procedure, duration of cardiopulmonary bypass, postoperative requirement for inotropic drugs and intra-aortic balloon pump (IABP), and postoperative indexes of organ dysfunction during ICU stay, were collected.

### 3.3. Statistical Analysis

Statistical analyses of the data were performed using SPSS for windows version 21.0 statistical software (SPSS Inc., Chicago, IL, USA). The categorical variables in the three groups were analyzed using chi-square test. In this study, parametric quantitative data were presented as the mean value and standard deviation and quantitative characteristics were described as the number and percentage for each category, for binary characteristics. There was also a 95% confidence interval (CI). Continuous variables were analyzed by one-way ANOVA. Receiver operating characteristic (ROC) curve was used to determine the cut-off point value of PTLC. The area under the ROC curve (AUC) showed this value. The value on the ROC curve was used to find out the maximum value of sensitivity and specificity as the cut-off point value. The percentages of sensitivity and specificity, as well as values of odds ratio (OR) with CI 95% were presented for the cut-off point value. Multivariate logistic regression analysis was used to determine the independent predictors of mortality. In this study, P Value ≤ 0.05 was considered statistically significant.

## 4. Results

Out of 1604 patients scheduled for cardiac surgery during six months, 1171 were included in the study. Patients underwent on-pump CABG (n = 674), valvular surgery (n = 363), and CABG + valvular surgery (n = 134). The study groups differed in the type of surgery (P = 0.0001). Of 1171 patients, 765 (65.3%) had PTLC > 1500 cell/µL, 380 (32.5%) had PTLC of 1001-1500 cell/µL, and 26 (2.2%) had PTLC ≤ 1000 cell/µL. Most of the patients who underwent CABG (n = 522) and valvular surgery (n = 201) were in the PTLC > 1500 cell/µL group, while most of the patients who underwent CABG + valvular surgery were in the PTLC of 1001-1500 cell/µL group. There were no statistically significant differences between the three groups regarding age and gender (Table 1). Analysis of demographic characteristics presented that the number of patients was significantly higher in the PTLC > 1500 cell/µL group than in the PTLC ≤ 1000 and PTLC of 1001-1500 cell/µL group (P = 0.0001). We found that PTLC ≤ 1000 cell/µL was more frequent in men than women. The number of diabetic patients was significantly higher in the PTLC of 1000-1500 cell/µL group than in the PTLC > 1500 cell/µL group (P = 0.0001). The lowest mean Ejection Fraction (EF) (32.5%) and the longest mean CPB time (230.04 minutes) were in the PTLC ≤ 1000 cell/µL (P < 0.001) group, compared with patients with higher PTLCs (Table 1).

**Figure 1.**
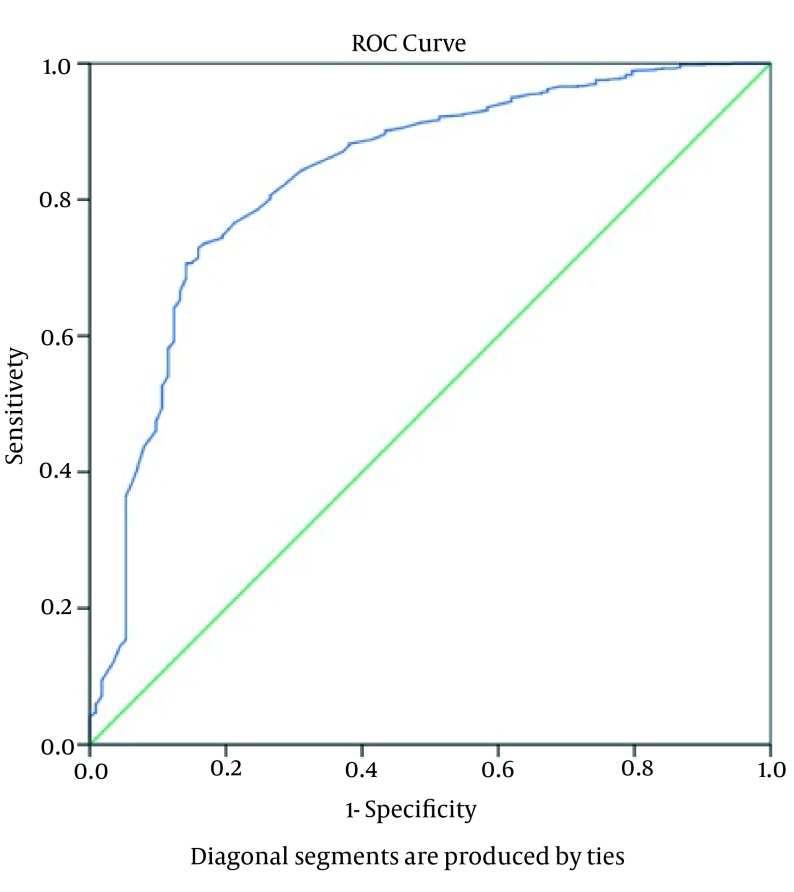
The Receiver Operating Characteristic Curve Showing the Mortality After Cardiac Surgery According to Preoperative Total Lymphocyte Count

**Figure 2.**
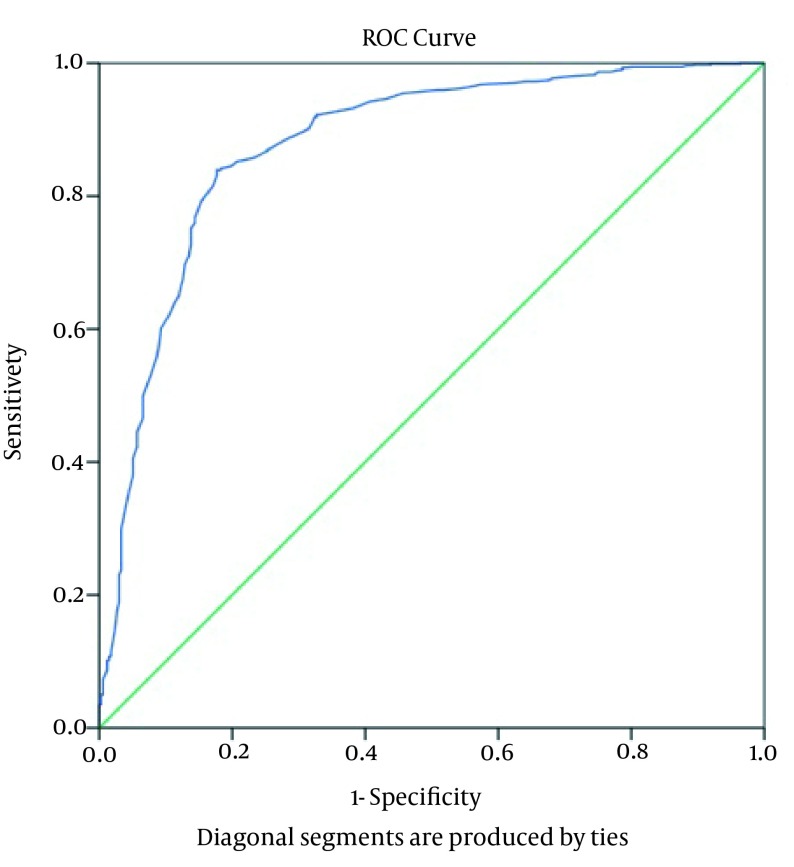
The Receiver Operating Characteristic Curve Showing Morbidity After Cardiac Surgery According to Preoperative Total Lymphocyte Count

Variance analysis of complications according to the groups (Table 2) showed that patients with PTLC of 1001-1500 cell/µL had the highest rate of complications (P = 0.0001). At the same time, the risk of dialysis-dependent ARF was significantly higher in the PTLC ≤ 1000 cell/µL group compared with the other two group (P = 0.0001). We found significant differences in the frequency of postoperative sternal wound infections and levels of need for inotropic support, and IABP was higher in the PTLC of 1001-1500 cell/µL group than in the PTLC > 1500 cell/µL group (P = 0.0001). In addition, patients with PTLC of 1000-1500 cell/µL had longer ICU stays and higher mortalities than the PTLC > 1500 cell/µL group (P = 0.0001) (Table 2). The ROC curves of PTLCs were obtained to determine their prognostic values for mortality, infectious complications, postoperative inotropic and IABP support, and dialysis-dependent ARF. The ROC curve results showed that PTLC = 1415 cells/µL is the cut-off point for prediction of mortality with 78.6% sensitivity and 76.2% specificity [AUC (95% CI) = 0.833 (0.790-0.876); P = 0.0001] (Figure 1 and Table 3). PTLC = 1492 cells/µL is the cut-off value for prediction of inotropic support requirement with 81.9% sensitivity and 89.3% specificity [AUC (95% CI) = 0.862 (0.836-0.887); P = 0.0001]. Cut-off point for prediction of IABP requirement is a PTLC equal to 1375 cells/µL with 81.3% sensitivity and 71.4% specificity [AUC (95% CI) = 0.827 (0.765-0.890); P = 0.0001]. The point of parameter separation for infectious complications is 1395 cells/µL with a sensitivity of 84% and a specificity of 86.8% [AUC (95% CI) = 0.890 (0.863-0.918); P = 0.0001]. The cut-off value for postoperative dialysis-dependent ARF is 1315 cells/µL with a sensitivity of 88% and a specificity of 90.9% [AUC (95% CI) = 0.963 (0.946-0.990); P = 0.000] and PTLC = 1485 cells/µL is the point of separation for prediction of all postoperative complications (morbidity) with a sensitivity of 84.5% and a specificity of 80.2% [AUC (95% CI) = 0.877 (0.853-0.900); P = 0.0001) (Table 3, Figure 2). According to the multivariate analysis (Table 4), postoperative inotropic and IABP support, dialysis-dependent ARF, and infectious complications, had significant influences on the mortality rate (Table 4).

**Table 3.** Receiver Operating Characteristic Parameters Showing the Value of Preoperative Total Lymphocyte Count on Postoperative Morbidity and Mortality^a^

	Cut-off point for PTLC	AUC (95% CI)	P Value	Sensitivity-Specificity, %
**Inotropic support**	≤ 1415	0.862 (0.836-0.887)	0.0001	81.90-89.30
**IABP support**	≤ 1375	0.827 (0.765-0.890)	0.0001	81.30-71.40
**Infectious complications**	≤ 1395	0.890 (0.863-0.918)	0.0001	84-86.80
**Dialysis-dependent ARF**	≤ 1315	0.963 (0.946-0.990)	0.0001	88-90.90
**Morbidity**	≤ 1485	0.877 (0.853-0.900)	0.0001	84.50-80.20
**Mortality**	≤ 1415	0.833 (0.790-0.876)	0.0001	78.60-76.20

^a^ Abbreviations: ARF, acute renal failure; AUC, area under the curve; IABP, intra-aortic balloon pump; PTLC, preoperative total lymphocyte count.

**Table 4.** Predictors of Mortality After Cardiac Surgery Using Multivariate Logistic Regression Analysis^a^

	OR (95% CI)	P Value
**PTLC**	0.99 (0.98-1.00)	0.037
**Inotropic support**	6.78 (3.47-13.25)	0.0001
**IABP support**	2.20 (1.02-4.70)	0.043
**Infectious complications**	3.01 (1.67-5.40)	0.0001
**Dialysis-dependent ARF**	2.11 (0.98-4.51)	0.055
**Operation type**	1.43 (1.04-1.97)	0.028

^a^ Abbreviations: ARF, acute renal failure; IABP, intra-aortic balloon pump; PTLC, preoperative total lymphocyte count.

## 5. Discussion

TLC provides an inexpensive, informative index for systemic inflammation and also helps to predict survival after open heart surgery (30). Several studies have shown that reduction in the lymphocyte count can be associated with a poor prognosis (8-12). We hypothesize that a reduced TLC acts as a marker for an ongoing nonspecific atherosclerotic inflammatory process. Lymphopenia has been associated with atherosclerosis progression and major adverse cardiac outcomes (10, 31, 32). In the present study, we also found that lymphopenia had strong relationship with lower EF as well as higher inotropic and IABP support of patients, postoperatively. There were significant differences among the mean EFs (%) of the three groups. The lowest EF was in the PTLC ≤ 1500 group, which was coincident with higher IABP insertion and inotropic requirement of the patients. Improved survival associated with a higher lymphocyte count has previously been observed in patients with stable coronary artery diseases (8, 9), those undergoing high-risk angioplasty (8), and ones with heart failures (20-23). Gennari et al. (33) and Dionigi et al. (34) revealed that the lymphocytopenia that occurred after major surgery, stemmed from neuroendocrine stress and led to cortisol production, which ultimately caused lymphocytopenia in the peripheral blood. Additionally, decreased lymphocytes observed after myocardial infarction (MI), have been linked to injuries connected with ischemia-reperfusion (IR) (35). The current data demonstrated that in patients undergoing cardiac surgery with CPB, the total lymphocyte count conveys powerful prognostic information (10). Similar to a prior study (9), we observed a close association between low preoperative TLC and prolongation of CPB time. We found that PTLC > 1500 cells/µL was associated with a significantly shorter CPB time, compared with the others two groups. In this study, low PTLC was associated with higher prevalence of diabetes (28), renal failure, and infection. This association coincided with the existing literature, describing these conditions as proinflammatory (36, 37). However, based on our study results, PTLC can be used as an independent, separate predictor of postoperative morbidity and mortality, which was not reported by other studies (9, 38, 39). On the other hand, it is obvious that malnutrition (24), severity of cardiovascular diseases, microcirculation impairment, and tissue hypoxia are causes of reducing the amount of PTLC and its functional disorders (26). Perhaps this can describe the finding that most of the patients in the PTLC ≤ 1000 cells/µL group had valvular heart diseases in the past. Analysis of baseline characteristics according to PTLC showed no correlations between age, sex and PTLC, while according to the data from other studies, elderly patients had a reduced PTLC and lymphocyte function (40, 41). Of baseline characteristics, BMI had a close relationship with low PTLC, which was similar to another study (42). Of five morbid events, either life threatening or potentially resulting in permanent functional disability (1, 43), we analyzed three morbid postoperative events according to PTLC, including mediastinitis, renal failure, and cardiovascular failure. In the present study, dialysis-dependent ARF was developed significantly more often in patients with a PTLC < 1500 cells/µL than in patients with a PTLC > 1500 cells/µL. This result was along with, but more distinguished than other studies (11, 44). There have been modifiable risk factors for sternal wound infection (45, 46). Since PTLC has the key role in the host defense against infection, we expected higher frequency of infectious complications including pneumonia and mediastinitis. However, the frequency of post-operative superficial wound infections was significantly higher in patients with lower PTLCs. The analysis of postoperative characteristics of surviving patients showed that patients with lower PTLCs required longer ICU stays than the ones with PTLC > 1500 cells/µL, and they had significantly higher morbidity and mortality rates. These findings were in accordance with other studies (47, 48).

In conclusion, PTLC provides a simple, inexpensive, informative, and producible index for systemic inflammation and helps to predict survival after cardiac surgery. In this study, we showed that reduced level of PTLC was directly correlated with mortality and inversely correlated with survival in the post-operative period. However, the large area under the ROC curve indicated that PTLC can be used as an independent, valuable prognostic criterion with high sensitivity and specificity for assessing the risk and evaluating the postoperative morbidity and mortality in cardiac surgery. Nevertheless, detailed mechanisms responsible for correlations between the preoperative PTLC and cardiovascular morbidity and mortality remain unknown; to illuminate this phenomenon, subsequent prospective studies are required.

### 5.1. Limitations

This was a single-center study with patients who were retrospectively enrolled from our database. Further studies are needed to explain the mechanism and evaluate the therapeutic applications of these findings. The use of morbidity and mortality as a primary end-point provides an objective measure of the outcome. On the other hand, no data were obtained about other important morbidities such as perioperative myocardial infarction or stroke. Data are also lacking about pre- and post-operative medications, heart failure functional class, and hemodynamic types of heart disease, which may have influenced the outcome of patients and possibly the PTLC itself.

Because of these limitations, some data that could influence the mortality rate were not involved in the present study. Despite these limitations, the current data demonstrated a clear relationship between PTLC and survival after cardiac surgery. Importantly, this prognostic utility is independent of other well-recognized individual risk factors. The unsolved problems listed previously showed that further investigations in this field are urgently needed.

## References

[1] Antunes PE, de Oliveira JF, Antunes MJ (2009). Risk-prediction for postoperative major morbidity in coronary surgery.. Eur J Cardiothorac Surg..

[2] Shahian DM, Edwards FH, Ferraris VA, Haan CK, Rich JB, Normand SL (2007). Quality measurement in adult cardiac surgery: part 1--Conceptual framework and measure selection.. Ann Thorac Surg..

[3] Hansson GK (2005). Inflammation, atherosclerosis, and coronary artery disease.. N Engl J Med..

[4] Choi JC, Bakaeen FG, Cornwell LD, Dao TK, Coselli JS, LeMaire SA (2012). Morbid obesity is associated with increased resource utilization in coronary artery bypass grafting.. Ann Thorac Surg..

[5] Thourani VH, Keeling WB, Kilgo PD, Puskas JD, Lattouf OM, Chen EP (2011). The impact of body mass index on morbidity and short- and long-term mortality in cardiac valvular surgery.. J Thorac Cardiovasc Surg..

[6] Corral L, Carrio ML, Ventura JL, Torrado H, Javierre C, Rodriguez-Castro D (2009). Is C-reactive protein a biomarker for immediate clinical outcome after cardiac surgery?. J Cardiothorac Vasc Anesth..

[7] Madjid M, Willerson JT (2011). Inflammatory markers in coronary heart disease.. Br Med Bull..

[8] Arbel Y, Finkelstein A, Halkin A, Birati EY, Revivo M, Zuzut M (2012). Neutrophil/lymphocyte ratio is related to the severity of coronary artery disease and clinical outcome in patients undergoing angiography.. Atherosclerosis..

[9] Azab B, Shariff MA, Bachir R, Nabagiez JP, McGinn JT Jr (2013). Elevated preoperative neutrophil/lymphocyte ratio as a predictor of increased long-term survival in minimal invasive coronary artery bypass surgery compared to sternotomy.. J Cardiothorac Surg..

[10] Bian C, Wu Y, Shi Y, Xu G, Wang J, Xiang M (2010). Predictive value of the relative lymphocyte count in coronary heart disease.. Heart Vessels..

[11] Korkmaz L, Kul S, Korkmaz AA, Akyuz AR, Agac MT, Erkan H (2012). Increased leucocyte count could predict coronary artery calcification in patients free of clinically apparent cardiovascular disease.. Turk Kardiyol Dern Ars..

[12] Lomivorotov V, Efremov S, Boboshko V (2011). Low preoperative total lymphocyte count as a predictor of poor outcome in adult cardiac surgery.. Crit Care..

[13] Bagger JP, Zindrou D, Taylor KM (2003). Leukocyte count: a risk factor for coronary artery bypass graft mortality.. Am J Med..

[14] Amaranto DJ, Wang EC, Eskandari MK, Morasch MD, Rodriguez HE, Pearce WH (2011). Normal preoperative white blood cell count is predictive of outcomes for endovascular procedures.. J Vasc Surg..

[15] Newall N, Grayson AD, Oo AY, Palmer ND, Dihmis WC, Rashid A (2006). Preoperative white blood cell count is independently associated with higher perioperative cardiac enzyme release and increased 1-year mortality after coronary artery bypass grafting.. Ann Thorac Surg..

[16] Madjid M, Fatemi O (2013). Components of the complete blood count as risk predictors for coronary heart disease: in-depth review and update.. Tex Heart Inst J..

[17] Li QQ, Lu ZH, Yang L, Lu M, Zhang XT, Li J (2014). Neutrophil count and the inflammation-based glasgow prognostic score predict survival in patients with advanced gastric cancer receiving first-line chemotherapy.. Asian Pac J Cancer Prev..

[18] Reddan DN, Klassen PS, Szczech LA, Coladonato JA, O'Shea S, Owen WF Jr (2003). White blood cells as a novel mortality predictor in haemodialysis patients.. Nephrol Dial Transplant..

[19] O'Daly BJ, Walsh JC, Quinlan JF, Falk GA, Stapleton R, Quinlan WR (2010). Serum albumin and total lymphocyte count as predictors of outcome in hip fractures.. Clin Nutr..

[20] Vaduganathan M, Ambrosy AP, Greene SJ, Mentz RJ, Subacius HP, Maggioni AP (2012). Predictive value of low relative lymphocyte count in patients hospitalized for heart failure with reduced ejection fraction: insights from the EVEREST trial.. Circ Heart Fail..

[21] Bekwelem W, Lutsey PL, Loehr LR, Agarwal SK, Astor BC, Guild C (2011). White blood cell count, C-reactive protein, and incident heart failure in the Atherosclerosis Risk in Communities (ARIC) Study.. Ann Epidemiol..

[22] Rudiger A, Burckhardt OA, Harpes P, Muller SA, Follath F (2006). The relative lymphocyte count on hospital admission is a risk factor for long-term mortality in patients with acute heart failure.. Am J Emerg Med..

[23] Battin DL, Ali S, Shahbaz AU, Massie JD, Munir A, Davis RC Jr (2010). Hypoalbuminemia and lymphocytopenia in patients with decompensated biventricular failure.. Am J Med Sci..

[24] Nishida T, Sakakibara H (2010). Association between underweight and low lymphocyte count as an indicator of malnutrition in Japanese women.. J Womens Health (Larchmt)..

[25] Bergquist J, Tarkowski A, Ewing A, Ekman R (1998). Catecholaminergic suppression of immunocompetent cells.. Immunol Today..

[26] Szigligeti P, Neumeier L, Duke E, Chougnet C, Takimoto K, Lee SM (2006). Signalling during hypoxia in human T lymphocytes--critical role of the src protein tyrosine kinase p56Lck in the O2 sensitivity of Kv1.3 channels.. J Physiol..

[27] Weigent DA (2013). Hypoxia and cytoplasmic alkalinization upregulate growth hormone expression in lymphocytes.. Cell Immunol..

[28] Kemal Demirağ M., Bedir A. (2013). Evaluation of preoperative neutrophil-lymphocyte ratio and platelet-lymphocyte ratio in patients undergoing major vascular surgery.. Turkish J Thoracic Cardiovasc Surg..

[29] Bhutta H, Agha R, Wong J, Tang TY, Wilson YG, Walsh SR (2011). Neutrophil-lymphocyte ratio predicts medium-term survival following elective major vascular surgery: a cross-sectional study.. Vasc Endovascular Surg..

[30] Gibson PH, Croal BL, Cuthbertson BH, Small GR, Ifezulike AI, Gibson G (2007). Preoperative neutrophil-lymphocyte ratio and outcome from coronary artery bypass grafting.. Am Heart J..

[31] Major AS, Fazio S, Linton MF (2002). B-lymphocyte deficiency increases atherosclerosis in LDL receptor-null mice.. Arterioscler Thromb Vasc Biol..

[32] Nunez J, Sanchis J, Bodi V, Nunez E, Mainar L, Heatta AM (2009). Relationship between low lymphocyte count and major cardiac events in patients with acute chest pain, a non-diagnostic electrocardiogram and normal troponin levels.. Atherosclerosis..

[33] Gennari R, Dominioni L, Imperatori A, Bianchi V, Maroni P, Dionigi R (1995). Alterations in lymphocyte subsets as prognosticators of postoperative infections.. Eur J Surg..

[34] Dionigi R, Dominioni L, Benevento A, Giudice G, Cuffari S, Bordone N (1994). Effects of surgical trauma of laparoscopic vs. open cholecystectomy.. Hepatogastroenterology..

[35] Arato E, Kurthy M, Sinay L, Kasza G, Menyhei G, Hardi P (2010). Effect of vitamin E on reperfusion injuries during reconstructive vascular operations on lower limbs.. Clin Hemorheol Microcirc..

[36] Dandona P, Aljada A, Bandyopadhyay A (2004). Inflammation: the link between insulin resistance, obesity and diabetes.. Trends Immunol..

[37] Miyamoto T, Carrero JJ, Stenvinkel P (2011). Inflammation as a risk factor and target for therapy in chronic kidney disease.. Curr Opin Nephrol Hypertens..

[38] Salis S, Mazzanti VV, Merli G, Salvi L, Tedesco CC, Veglia F (2008). Cardiopulmonary bypass duration is an independent predictor of morbidity and mortality after cardiac surgery.. J Cardiothorac Vasc Anesth..

[39] Ferguson TJ, Hammill BG, Peterson ED, DeLong ER, Grover FL, S. T. S. National Database Committee (2002). A decade of change--risk profiles and outcomes for isolated coronary artery bypass grafting procedures, 1990-1999: a report from the STS National Database Committee and the Duke Clinical Research Institute. Society of Thoracic Surgeons.. Ann Thorac Surg..

[40] Chandra RK (2002). Nutrition and the immune system from birth to old age.. Eur J Clin Nutr..

[41] Yazdanian F, Azarfarin R, Aghdaii N, Jalali-Motlagh S, Faritous Z, Alavi M (2012). Relationship Between Gender and In-Hospital Morbidity and Mortality After Coronary Artery Bypass Grafting Surgery in an Iranian Population.. Res Cardiovasc Med..

[42] Koochemeshki V, Amestejani M, Salmanzadeh HR, Salehi Ardabili S (2012). The Effect of Obesity on Mortality And Morbidity after Isolated Coronary Artery Bypass Grafting Surgery.. Int Cardiovasc Res J..

[43] Simon C, Luciani R, Capuano F, Miceli A, Roscitano A, Tonelli E (2007). Mild and moderate renal dysfunction: impact on short-term outcome.. Eur J Cardiothorac Surg..

[44] Lomivorotov VV, Efremov SM, Boboshko VA, Leyderman IN, Lomivorotov VN, Cheung AT (2011). Preoperative total lymphocyte count in peripheral blood as a predictor of poor outcome in adult cardiac surgery.. J Cardiothorac Vasc Anesth..

[45] Lu JC, Grayson AD, Jha P, Srinivasan AK, Fabri BM (2003). Risk factors for sternal wound infection and mid-term survival following coronary artery bypass surgery.. Eur J Cardiothorac Surg..

[46] Hassan M, Smith JM, Engel AM (2006). Predictors and outcomes of sternal wound complications in patients after coronary artery bypass graft surgery.. Am Surg..

[47] De Cocker J, Messaoudi N, Stockman BA, Bossaert LL, Rodrigus IE (2011). Preoperative prediction of intensive care unit stay following cardiac surgery.. Eur J Cardiothorac Surg..

[48] Acanfora D, Gheorghiade M, Trojano L, Furgi G, Pasini E, Picone C (2001). Relative lymphocyte count: a prognostic indicator of mortality in elderly patients with congestive heart failure.. Am Heart J..

